# Angular Dispersion of Free-Electron-Light Coupling in an Optical Fiber-Integrated Metagrating

**DOI:** 10.1021/acsphotonics.3c01574

**Published:** 2024-03-02

**Authors:** Matthias Liebtrau, Albert Polman

**Affiliations:** Center for Nanophotonics, NWO-Institute AMOLF, Science Park 104, 1098 XG Amsterdam, The Netherlands

**Keywords:** free-electron-light coupling, Smith−Purcell radiation, cathodoluminescence, metasurface, nanophotonics, optical fiber

## Abstract

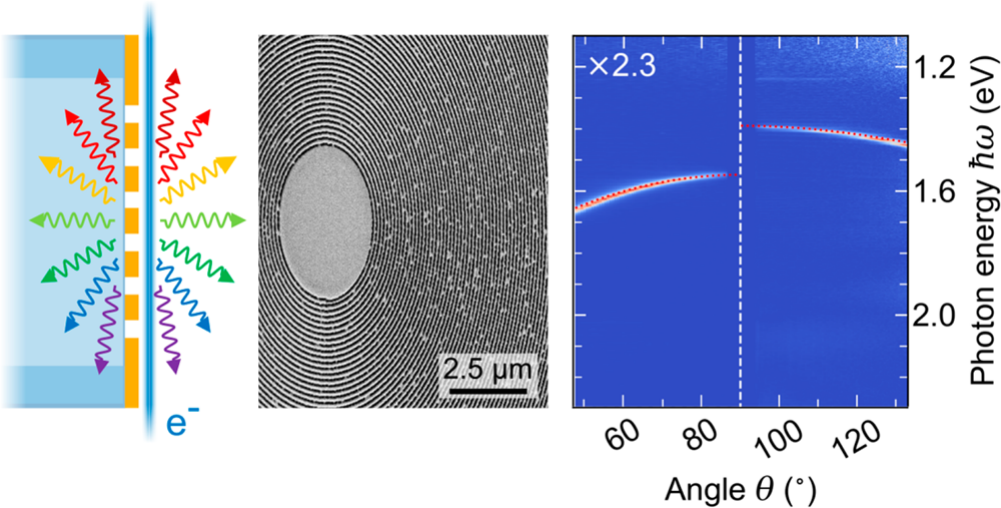

Free electrons can couple to optical material excitations on nanometer-length and attosecond-time scales, opening-up unique opportunities for both the generation of radiation and the manipulation of the electron wave function. Here, we exploit the Smith–Purcell effect to experimentally study the coherent coupling of free electrons and light in a circular metallo-dielectric metagrating that is fabricated onto the input facet of a multimode optical fiber. Using hyperspectral angle-resolved (HSAR) far-field imaging inside a scanning electron microscope, we probe the angular dispersion of Smith–Purcell radiation (SPR) that is simultaneously generated in free space and inside the fiber by an electron beam that grazes the metagrating at a nanoscale distance. Furthermore, we analyze the spectral distribution of SPR that is emitted into guided optical modes and correlate it with the numerical aperture of the fiber. By varying the electron energy between 5 and 30 keV, we observe the emission of SPR from the ultraviolet to the near-infrared spectral range, and up to the third emission order. In addition, we detect incoherent cathodoluminescence that is generated by electrons penetrating the input facet of the fiber and scattering inelastically. As a result, our HSAR measurements reveal a Fano resonance that is coupled to a Rayleigh anomaly of the metagrating, and that overlaps with the angular dispersion of second-order SPR at 20 keV. Our findings demonstrate the potential of optical fiber-integrated metasurfaces as a versatile platform to implement novel ultrafast light sources and to synthesize complex free-electron quantum states with light.

## Introduction

In free space, the net exchange of energy between a free electron and a photon is usually forbidden due to the violation of momentum conservation.^1^ In the vicinity of nanostructures, however, the formation of a localized optical field can mediate their interaction.^2^ In recent years, this phenomenon has been widely explored to probe optical material properties on nanoscale-length and (sub-) femtosecond-time scales, to generate coherent radiation over an ultrabroad spectral range, and to manipulate the wave function of free electrons in both space and time.^3−6^ To further advance these developments, new insights into the interaction of free electrons, light, and matter and how to control this phenomenon are of great interest.

The interaction of free electrons and light in nanomaterials can occur both as a spontaneous and as a stimulated process.^3,7−10^ On the one hand, the evanescent electromagnetic field of the electron itself can drive optical material excitations across a broad spectral range from the extreme ultraviolet (XUV) to the far-infrared (IR).^3,11^ During this process, the electron undergoes a characteristic energy loss that is correlated with the local density of optical states (LDOS) along the electron trajectory.^3,12,13^ The radiative component of the LDOS gives rise to the emission of cathodoluminescence (CL),^13,14^ the properties of which can be probed experimentally using angle- and polarization-resolved CL spectroscopy.^15−17^ On the other hand, the near field of a material that is excited by an external optical pump field can induce discrete electron energy-gain and energy-loss transitions by mediating the stimulated absorption and emission of photons at the pump photon energy.^1,7,8^ In photon-induced near-field electron microscopy (PINEM),^18^ the resulting modulation of the electron energy spectrum serves as a direct measure of the electron–photon coupling strength, enabling spectrally-,^19^ spatially-,^9,20−22^ temporally-,^23−30^ polarization-,^9,18,20,31^ and phase-resolved near-field measurements.^25,28−30,32^ Furthermore, this stimulated interaction mechanism can be harnessed to manipulate the phase and amplitude of the electron wave function,^22,33−35^ facilitating the synthesis of ultrashort free-electron wave packets,^23−25,28,36^ transversely modulated electron beams,^37,38^ and complex free-electron quantum states.^39,40^

For free electrons and photons to couple, the optical field that mediates their interaction has to contain a spatial-frequency Fourier component that remains in phase with the electron throughout the interaction—a condition known as electron-light-phase matching.^2^ As a result, the interaction is highly dependent on the electron velocity, with slow electrons favoring high spatial frequency components in tightly concentrated optical fields, while fast electrons preferentially couple to more extended field distributions.^9,41−43^ Nevertheless, a localized optical field is naturally composed of a broad distribution of spatial frequency components. Hence, the fraction of electromagnetic energy that mediates the interaction is typically small. In contrast, systems that comprise a large number of nanostructures permit to shape the coupling of free electrons and light, much like optical metasurfaces that permit to tailor optical fields in real space and the Fourier domain.^44−49^

A well-known example for the coupling of free electrons and light in periodic systems is the so-called Smith–Purcell (SP) effect.^50^ The SP effect arises if an electron polarizes the system along a grazing trajectory, leading to the emission of broadband radiation into discrete diffraction orders *m*. Assuming a grating of pitch *p* that is excited orthogonal to the rulings, the angular dispersion relation of this radiation can be written as^50^
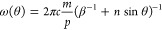
1with ω as the frequency, β = *v*/*c* as the electron velocity in units of the speed of light in vacuum *c*, θ as the emission angle, and *n* as the refractive index of the surrounding medium. Here, the emission angle is defined relative to the plane normal to the grating and the electron trajectory, with θ = −90° corresponding to the direction of propagation of the electron. The light waves that couple to the electron in the near field of the grating carry spatial frequencies that are determined by the wave vector of the outgoing photons and the harmonic orders of the reciprocal lattice constant.^51,52^ Thus, for each harmonic order *m*, only a single combination of emission angle θ and frequency ω fascilitates electron-light-phase matching as addressed above.

Since its discovery several decades ago,^50^ the SP effect has been extensively explored in the literature, both theoretically and experimentally.^53^ The recent advent of optical metasurfaces offers great potential to control this phenomenon by tailoring the spectrum^43,54−56^ and the angular distribution,^52,57,58^ as well as the polarization of SPR.^52,58−60^ Similarly, the inverse SP effect^61−65^ can be harnessed in metasurfaces to mediate the exchange of energy between free electrons and an optical pump field, as in PINEM. However, interfacing such structures with suitable optics for the extraction or injection of radiation, poses another experimental challenge. A few years ago, the authors of refs (66 and 67) demonstrated an approach based on metallic nanogratings that are fabricated onto the input facet of an optical fiber to simultaneously generate and couple SPR into guided optical modes. This enabled the extraction of SPR through the fiber from the visible (VIS) to the ultraviolet (UV) spectral range. However, the angular features and, hence, the dispersion relation of the coupling between free electrons and light in such a geometry have not been studied yet.

In this work, we apply hyperspectral angle-resolved (HSAR) far-field imaging inside a scanning electron microscope (SEM)^16,17^ to probe the angular dispersion of the SP effect in a circular metallic metagrating that is fabricated onto the cleaved input facet of a multimode optical fiber. Our measurements reveal the characteristic signature of SPR that is generated on both the free-space side and the fiber-core side of the metagrating by exploiting radiation that leaks out of the fiber. Furthermore, we measure the spectral distribution of SPR that couples to guided optical modes and correlate it with the numerical aperture (NA) of the fiber. By varying the electron energy from 5 to 30 keV, we observe the emission of SPR across the entire UV to NIR spectral range and up to the third diffraction order. Additionally, we find that electrons penetrating the input facet of the fiber generate incoherent defect cathodoluminescence that couples to a Rayleigh anomaly of the metagrating. As a result, our HSAR measurements reveal a Fano resonance that overlaps with the dispersion relation of second-order SPR at 20 keV. The analysis of our data is supported by an analytical model that allows us to efficiently describe and distinguish the spectral and angular features of SPR and the incoherent signal contributions.

The findings presented in this work are of great relevance to the design of novel electron-light coupling geometries that harness the SP effect in both light-collection and optical-pump configurations. The simultaneous observation of SPR and a Fano resonance inspires further thoughts on how metasurfaces can be used to combine, enhance, and control the coupling of free electrons and light by exploiting collective nanophotonic excitations such as lattice resonances.^55,68,69^ In addition, similar geometries based on single-mode fibers may enable the correlated spectroscopic detection of electrons and photons, potentially revealing new insights into the electron–light–matter interaction and opening up a promising route toward entangled electron–photon states for quantum applications.^70^

## Methods

**Figure 1:**
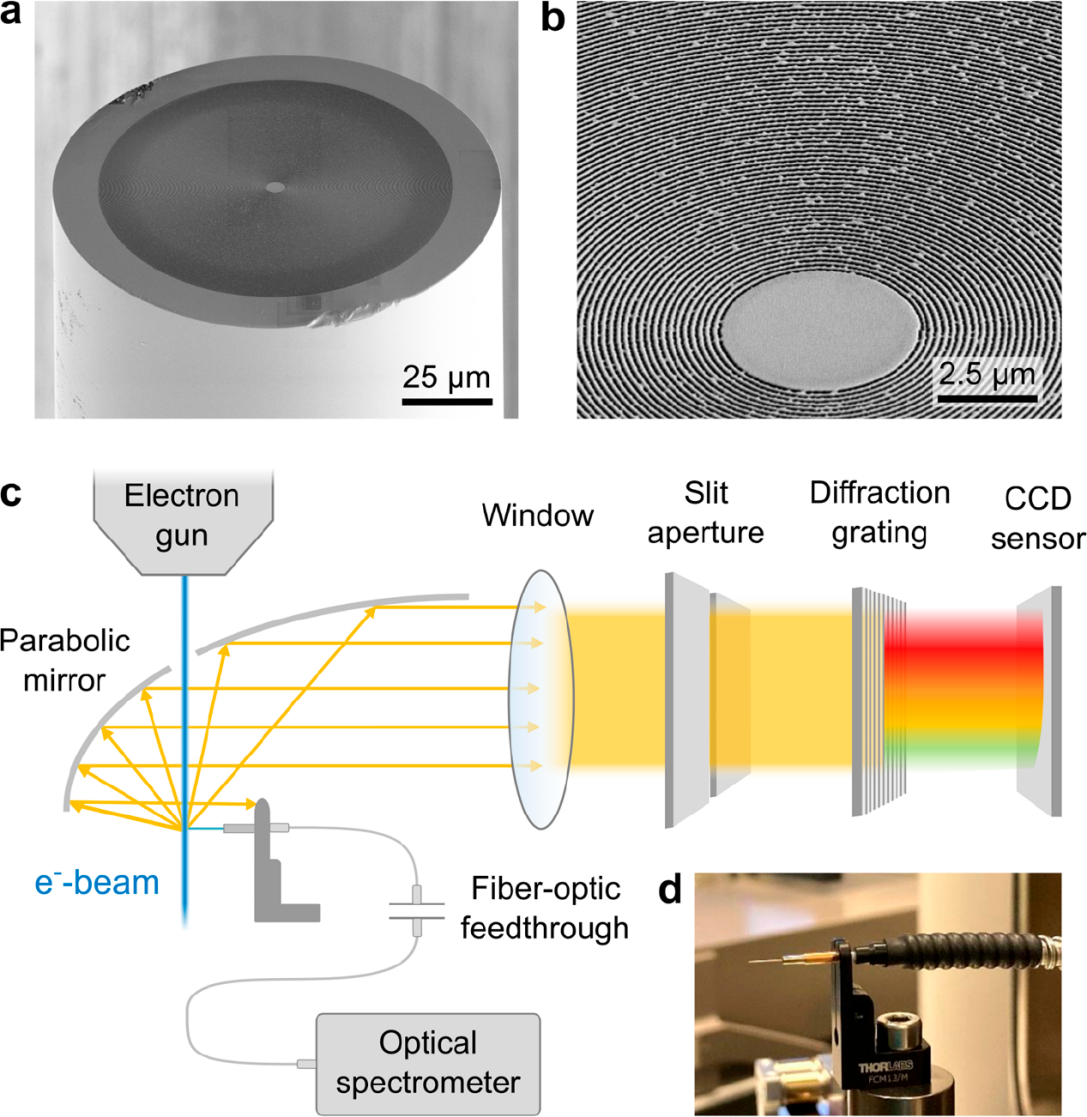
(a) SEM micrograph of a circular metallic metagrating that is fabricated onto the cleaved input facet of a multimode optical fiber. (b) Close-up view of concentric rings in the center of the pattern in (a) with a 200 nm radial pitch. (c) Simplified schematic overview of the experimental setup that is used for the observation of the SP effect through the fiber and in the far-field. The electron beam of an SEM is aligned with the metagrating along a grazing trajectory, simultaneously generating SPR in free space and inside the fiber. (d) Photograph of the patterned fiber protruding from a stainless-steel ferrule connector that is coupled to a standard multimode fiber-optic patch chord. The sample is mounted on a motorized stage that can be tilted to achieve an optimum beam-sample alignment.

Using focused ion beam milling (FEI Helios NanoLab 600, Thermo-Fisher Scientific, Inc.), a circular metallic metagrating was fabricated onto the input facet of a multimode optical fiber (numerical aperture NA = 0.22, 105 μm diameter silica core, 10 μm cladding, CFML21L05, Thorlabs, Inc.). To this end, the fiber was coated with a 5 nm thick Cr adhesion layer and a 45 nm thick Au film that were applied by sputter deposition (EM ACE600, Leica, Inc.). An SEM micrograph of the resulting structure is shown in Figure 1a. The metagrating comprises 237 concentric rings with a nominal radial pitch of 200 nm and a maximum diameter of 100 μm. The rings are separated by approximately 100 nm wide gaps that extend into the silica substrate of the fiber core. In the center, a 5 μm diameter circular patch is retained as an alignment marker. A close-up view on the metagrating in Figure 1b shows a few milling defects that result from the crystallographic disorder of the metal coating.^71^ However, as demonstrated in previous work,^56^ minor fabrication imperfections do not cause a substantial distortion of SPR as long as the desired periodicity of the pattern remains.

Figure 1c shows a simplified overview of the experimental setup that is used for the observation of the SP effect both in the far field and through the fiber. We operate a conventional SEM instrument (FEI Quanta 650 FEG, Thermo-Fisher Scientific Inc.) that is equipped with an optical setup for hyperspectral angle-resolved (HSAR) far-field detection of free-electron radiation (SPARC Spectral, Delmic B.V.).^16,17^ The setup comprises an off-axis half-parabolic mirror that is focused onto the end facet of the fiber from above, while the electron beam passes to the sample through a narrow aperture. Light that is collected within a narrow azimuthal angular range around the symmetry plane of the mirror is transmitted through a slit aperture and dispersed by a diffraction grating. Subsequently, the light is projected onto a two-dimensional silicon sensor array, with the horizontal and the vertical sensor axes resolving the spectral and the zenithal angular information, respectively. The output signal of the fiber is fiber-coupled into an external optical spectrometer with a liquid-nitrogen-cooled silicon sensor (Spectra Pro 2300i, Princeton Instruments, Inc.). For this purpose, the fiber is fitted with a stainless-steel ferrule connector that is mated to a conventional multimode optical fiber with a matching NA and core diameter as pictured in Figure 1d. The other end of this fiber is attached to a fiber-optic vacuum feedthrough, guiding the light to the spectrometer outside the SEM vessel.

The angular light collection range of the parabolic mirror spans from θ_min_ ≈ 11° at the mirror apex to θ_max_ ≈ 133° at the opening, with θ = 0° corresponding to light that is collected normal to the metagrating. However, in our experiments the sample holder introduces a shadow that effectively shifts the lower light collection limit to θ_min_ ≈ 46–47°, depending on the precise vertical mirror–sample alignment. In addition, the aperture that transmits the electron beam covers the angular range from θ = 86.4 to 93.4°. For the acquisition of HSAR data in the NIR spectral range, a long-pass filter with a cutoff energy of 2.48 eV is inserted in front of the slit aperture to avoid spectral overlap between the first and second diffraction order of the diffraction grating. This measure eliminates signal artifacts down to half the cutoff photon energy of 1.24 eV. Similarly, a long-pass filter with a cutoff energy of 1.97 eV is inserted at the sensor input of the fiber-coupled spectrometer. To retrieve absolute photon emission probabilities per incident electron, the HSAR data are calibrated using a reference measurement of transition radiation that is excited at the surface of a monocrystalline Al sample. Further details on the underlying spectral and angular data calibration procedure are provided in the Supporting Information of ref (52). To correct for the spectral system response function of the fiber-coupled spectrometer, we use a halogen white-light lamp (AvaLight-HAL-CAL-MINI, Avantes B.V.) as a reference source.

Our experiments are performed using an electron beam with kinetic energies in the range of 5 to 30 keV (beam current 0.8–1.6 nA). The beam is focused to a spot size of a few tens of nanometers and aligned with the metagrating along a grazing trajectory. To this end, the sample is mounted on a mechanical alignment stage that can be tilted, rotated, and translated in three dimensions. Using HSAR imaging, an optimum beam-sample alignment is achieved by minimizing the spectral line shape of the SPR signal for a fixed emission angle. In view of an alignment uncertainty of ∼1–2 mrad, the electron beam is chosen to gradually approach the structure under a very small angle rather than diverting from it. Thus, we ensure efficient generation of SPR at a nanoscale distance over a maximum interaction range.

## Results

### Coherent and Incoherent Signal Contributions

**Figure 2:**
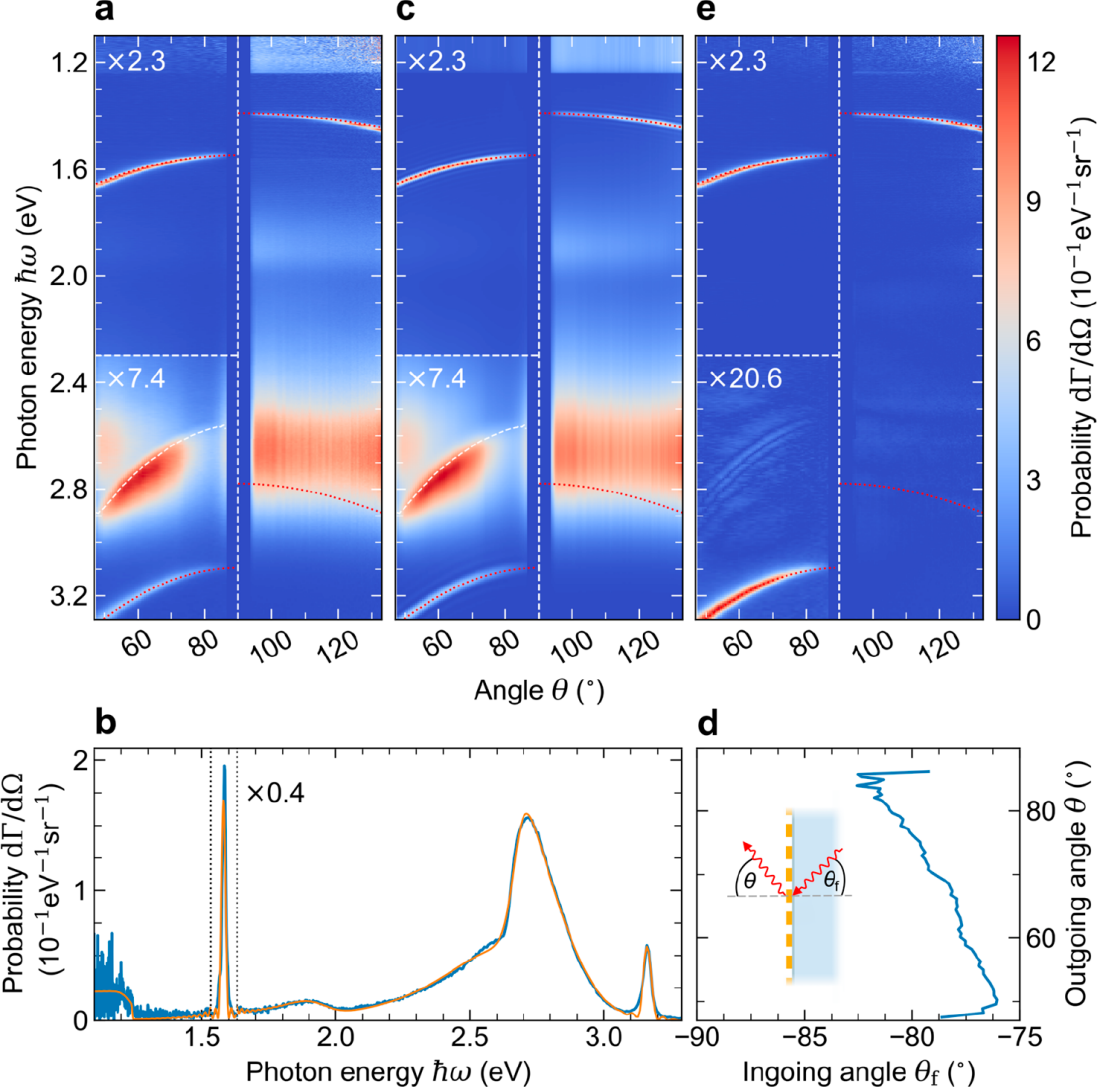
(a) HSAR measurement of far-field radiation induced by a 30 keV electron beam that is aligned with the center of the metagrating along a grazing trajectory. The data reveal the coherent excitation of SPR as well as the generation of incoherent defect cathodoluminescence that occurs due to the penetration of electrons into the fiber. The dotted-red lines represent the expected theoretical dispersion curves of first- and second-order SPR that is observed from both the free-space and the fiber-core sides of the metagrating at angles smaller and larger than θ = 90°, respectively (cf. eqs 1 and 2). The white-dashed line represents a model fit to the angular dispersion relation of a Fano resonance that occurs due to a Rayleigh anomaly of the metagrating (cf. eq 3). All curves are retrieved assuming a grating pitch of *p* = 198.0 nm, and a refractive index of the fiber core of *n* = 1.46. (b) Spectral cross cut through the data in (a) at an angle of θ ≈ 66°. The measurement (blue curve) is superimposed with a fitted model curve (orange) that is used to separate the coherent and incoherent signal contributions. The signal of first- and second-order SPR corresponds to the two narrow peaks at 1.6 and 3.2 eV, while the Fano resonance is observed by the large asymmetric peak with a maximum near 2.7 eV. (c) Collection of analytical model curves fitted to each angular data slice of the measurement in (a). (d) Fitted correlation between the in- and outgoing angles of photons that couple to the Rayleigh anomaly of the metagrating as illustrated in the inset. (e) Absolute value of the SPR signal as retrieved by subtracting the incoherent signal contributions of the model curves in (c) from the experimental data in (a).

Figure 2a shows an HSAR measurement acquired using a 30 keV electron beam that grazes the center of the metagrating. The data are compiled from two consecutive measurements in the UV–vis and NIR spectral ranges that are merged at a photon energy of 1.56 eV. We plot the differential photon emission probability dΓ/dΩ per unit photon energy ℏω and per unit solid angle Ω. On both sides of the metagrating, several striking features are observed that can be divided into coherent and incoherent radiation phenomena as discussed in detail below.

First, we consider two narrow curved bands with spectral onsets near 1.55 and 3.10 eV on the free-space side of the metagrating at angles θ < 90°. These bands can be readily identified as the emission of first- and second-order SPR. As shown by the dotted-red curves, the data are in good agreement with the theoretical dispersion relation predicted by eq 1 for *n* = 1.0, *m* = 1 and 2, and *p* = 198.0 nm. Turning our attention to angles θ > 90°, we find another similar band with an opposite curvature and a spectral onset close to 1.4 eV. Intriguingly, this observation demonstrates the emission of first-order SPR into the fiber by electrons that evanescently couple to the backside of the metagrating. The observed light leaks through the cladding layer and is subsequently refracted at the fiber/vacuum interface, leading to an effective dispersion relation of the form
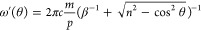
2where the emission angle is shifted according to Snell’s law. The resulting dispersion curve is in excellent agreement with the data for *n* = 1.46, *m* = 1, and *p* = 198.0 nm(dotted-red line). For reference, we also draw the second-order curve (*m* = 2), which, however, cannot be correlated to a measured signal. The red shift between SPR inside the fiber and in free space is determined by the refractive index of the silica core, as described by eq 1. Incidentally, the transmissivity of the metal coating is estimated to be of the order of a few percent with the skin depth of 13 nm in Au and 20 nm in Cr at a photon energy of 1.4 eV. However, on the sidewalls of the fiber the coating might be thinner and less uniform, therefore being potentially more transparent.

Next, we turn our attention to two flat incoherent emission bands on the backside of the metagrating (θ > 90°), each of which feature a broad spectral distribution around photon energies of 1.9 and 2.7 eV. Due to the above-mentioned alignment imperfections and the convergence angle of the electron beam (1–2 mrad), these bands can be explained by electrons that penetrate the input facet of the fiber and scatter inelastically, thereby generating radiative defect excitations in the silica network. In good correspondence with previous reports in the literature, the low- and high-energy emission bands can be assigned to nonbridging oxygen hole centers (NBOHCs) and oxygen deficient centers (ODCs), respectively.^72,73^ Notably, the signal amplitude of the ODC band is similar in magnitude to that of the first-order SPR bands. As a result, spectral overlap between the first and the second diffraction order of the ODC band in the HSAR imaging setup causes an artificial background signal below 1.24 eV, as explained in Methods.

On the free-space side of the metagrating (θ < 90°), the NBOHC and the ODC bands are also observed, however, at a much-reduced intensity. Instead, we find a strongly dispersive feature that dominates the ODC spectral range between 2.55 and 2.90 eV. For reference, Figure 2b shows a spectral cross cut through the data at an angle of θ ≈ 66°, revealing a large asymmetric peak with a maximum near 2.7 eV that is superimposed on the ODC band. Interestingly, we can explain this observation by a Fano resonance that occurs due to the coupling of the incoherent radiative excitations to a Rayleigh anomaly^74^ of the metagrating.^75,76^ Assuming an effective index *n*_eff_ = *n* sin θ_f_ for light propagating along the silica/metagrating interface, the latter follows an angular dispersion relation of the form
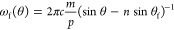
3with ω_f_ as the photon frequency, *m* as the diffraction order, and θ_f_ and θ as the incident and outgoing angles of the diffracted photons, respectively. Since the free-space wavelength of the observed light is larger than the grating pitch, we note that only in- and outgoing angles of opposite sign are supported by the metagrating, corresponding to the *m* = 1 diffraction order. To exclude the contribution of a coherent process, a reference measurement is performed that shows the Fano resonance is also observed when the electron beam impacts onto the fiber without interacting with the metagrating. In addition, the dispersion of the Fano peak is found to be independent of the electron energy, as further demonstrated below, in stark contrast to the dispersion relation of SPR (cf. eq 1).

### Analytical Signal Modeling and Filtering

To facilitate an in-depth analysis and interpretation of our experimental data, we resort to an analytical modeling approach that allows us to efficiently separate the coherent and incoherent signal contributions. The latter are captured by combining a superposition of two exponentially modified Gaussian distributions with a Fano model, as detailed in Section S1 of the Supporting Information. The SPR signal is modeled assuming that the electron beam propagates at a constant grazing distance *d* to the metagrating. Thus, as derived in Section S2 of the Supporting Information, the spectral line shape of the *m*th SPR emission order can be approximated as
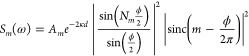
4with sinc(*x*) = sin(π*x*)/(π*x*) as the normalized sinc function, *A*_*m*_ as the signal amplitude and *N*_*m*_ as the number of unit cells that couple to the far field. The leading term in this expression describes an exponential decay of the signal strength with the grazing distance *d*, where  is the frequency-dependent decay constant. This behavior follows from the evanescent decay of the Fourier wave in the near field of the metagrating that mediates the excitation of SPR, as discussed above and described in more detail in the Supporting Information of ref (52). The remaining two terms can be interpreted in close analogy to the diffraction of light by an array of *N*_*m*_ slits. Here, the middle term accounts for the interference between the slits, and the last term captures the diffraction pattern of an individual slit, i.e., a single unit cell of the metagrating. The SPR dispersion relation enters both terms through the parameter , which we alternatively write as  to take into account the refraction of SPR at the fiber/vacuum interface (cf. eq 2 for θ > 90°). Notably, eq 4 shows that the spectral line shape of the SPR signal is primarily determined by the number of unit cells *N*_*m*_ that couple to the electron beam. Due to minor alignment imperfections and the intrinsic beam divergence, however, this value is typically lower than the nominal number of unit cells in the metagrating. Therefore, we estimate an effective number of unit cells *N*_*m*_ to describe the signal of each SPR order separately. The grazing distance *d* has a comparably small effect on the spectral line shape of the SPR signal, but mostly determines the signal amplitude, which is absorbed in the fitting parameter *A*_*m*_. Therefore, we chose an estimated grazing distance of *d* ≈ 10 nm (roughly the scale of the electron beam probe width at 30 keV).

The orange curve in Figure 2b shows a least-squares fit of the full analytical model to the spectrum at θ ≈ 66°, assuming *n* = 1.46, *m* = 1 and 2, and *p* = 198.0 nm. We find excellent agreement of the Fano model with the large asymmetric peak at 2.7 eV and the incoherent background due to the NBOHC and ODC bands. The signal of first- and second-order SPR is observed as two narrow peaks with maxima at approximately 1.58 and 3.16 eV, respectively. Their corresponding line widths are captured assuming an excitation of *N*_1_ = 80 and *N*_2_ = 40 unit cells in the metagrating. The large difference between these values can be ascribed to the exponential decay of the SPR excitation efficiency with the photon energy and thus the SPR order, as determined by the decay constant κ in eq 4.

Figure 2c shows a complete set of model fits to all spectra within the angular data range of the measurement in Figure 2a. The curves are based on the same assumptions for the parameters *n*, *m*, *p*, *d*, *N*_1_, and *N*_2_, as specified above, while all the remaining parameters are fitted separately. The model consistently captures the spectral and angular features of both the coherent and incoherent radiation phenomena. For reference, we superimpose the dispersion curves of first- and second-order SPR, as defined by eqs 1 and 2 (red-dotted curves). Moreover, we plot the dispersion relation of the Fano resonance in Figure 2a,c (white-dashed curve) using the parameters ω_f_ and θ_f_ as derived from our fitting procedure. Following the signal intensity along this curve, we find that the Fano peak gradually varies in amplitude, reaching a maximum visibility in the angular range between θ ≈ 55° and 70°. We anticipate that this intensity distribution is correlated with both the spectral distribution of the ODC band and the angular distribution of the photons that are generated inside the fiber. Also, the precise angle-dependent diffraction efficiency of the metagrating is expected to determine the signal amplitude. For reference, Figure 2d shows the correlation between the fitted incident angle θ_f_ and the corresponding outgoing angle θ, with the inset illustrating their relative orientation. As a prevalent trend, we find an increase of the enclosed angle δθ = θ – θ_f_ with decreasing energy of the diffracted photons ℏω_f_, in good agreement with the typical dispersion of a diffractive process. Figure 2e shows the SPR signal, as retrieved by subtracting the fitted incoherent signal from the experimental data in Figure 2a. We plot the absolute value of this difference to best represent the correspondence between the fit and the measurement. As clearly seen by the second-order SPR band, our filtering procedure excellently isolates the SPR signal, facilitating a detailed analysis of free-electron-light coupling via the metagrating.

### Analysis of Free-Electron-Light Coupling via the Metagrating

**Figure 3:**
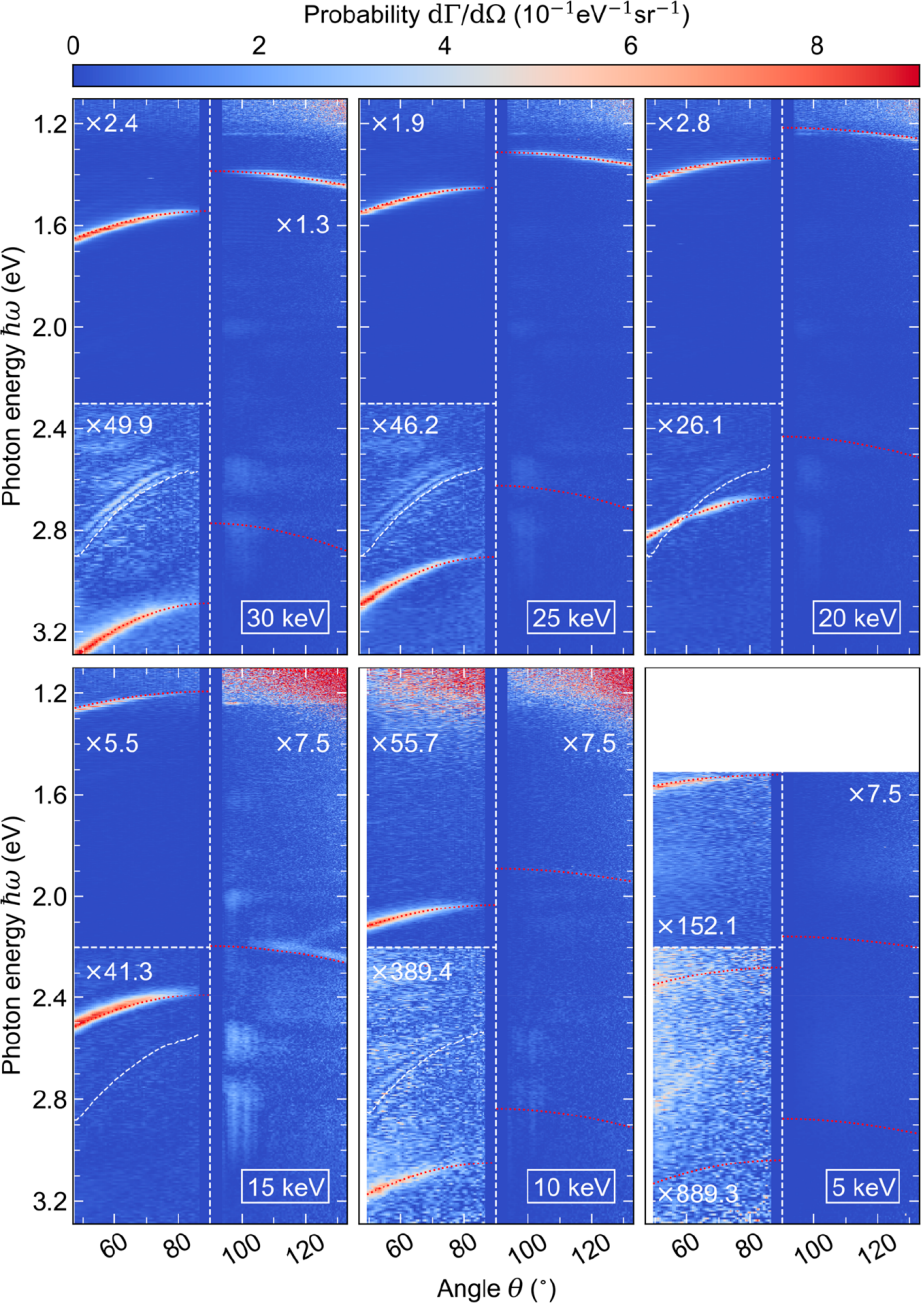
Measured dispersion of the SPR signal as a function of the electron energy. All signal distributions, except for the data at 5 keV, are processed using the same filtering procedure as described in Figure 2. The red-dotted lines show the calculated dispersion curves of all SPR orders within the spectral range of observation (eqs 1 and 2). The curves are derived assuming grating pitches *p* between 198.0 and 199.0 nm. In addition, the fitted dispersion relation of the Fano resonance (eq 3) is superimposed with the panels from 30 to 10 keV. At 20 keV, this dispersion overlaps with that of the second-order SPR band in free space.

Figure 3 shows the effect of the electron energy on the angular dispersion of SPR. In six consecutive measurements, the electron energy is lowered from 30 to 5 keV, corresponding to a reduction in the electron velocity from β ≈ 0.33 to β ≈ 0.14. After the measurement at 15 keV, the vertical position of the fiber end-facet was realigned relative to the parabolic light collection mirror. Therefore, the lower cutoff angle in the measurements at 5 and 10 keV is slightly increased by approximately 1.5°. The SPR signal is separated from the incoherent background contributions using the same filtering procedure as described above. To this end, a constant grating pitch *p* is chosen between 198.0 and 199.0 nm, assuming a small error margin of Δ*p* = 1 nm. Moreover, to account for differences in beam quality between the different electron energies, the parameter *N*_*m*_ is estimated separately. At 5 keV, the signal-to-noise ratio is too low to reasonably fit the data, however, the incoherent signal contributions are negligible due to little penetration of electrons into the fiber. For reference, all measurements are overlaid with the dispersion curves of the SPR orders that fall within the spectral range of observation, as calculated from eqs 1 and 2. In addition, the fitted dispersion relation of the Fano resonance is represented by the white-dashed line in each panel, confirming that the phenomenon is an incoherent process that does not shift with the electron energy (as opposed to SPR).

On the free-space side of the metagrating (θ < 90°), we observe first- and second-order SPR bands at electron energies from 15 to 30 keV. The measurements at 5 and 10 keV reveal second- and even third-order SPR bands. On the silica side (θ > 90°), the first-order SPR band is detected at 20 keV and above, while at 15 keV, a faint signature of the second order is retrieved. Overall, the measurements demonstrate a consistent red-shift of the SPR signal with decreasing electron energy, in good quantitative agreement with the theory. Comparing the signal amplitudes, the excitation strength of first-order SPR in free space is approximately 1 and 2 orders of magnitude larger than those of the second and the third orders, respectively. These ratios follow from the intensity distribution of the Fourier waves that couple to the electron in the near-field of the metagrating. Modeling the field profile along the electron trajectory by a rectangular wave with an effective duty cycle η, the relative intensity of the Fourier components can be approximated as sinc^2^(η*m*), where *m* is the harmonic order of the reciprocal lattice constant 2π/*p*. For *η* = 0.5, only odd orders *m* can exist in the grating, hence, there would be no generation of second-order SPR. For *η* = 0.7, the intensities of the second and third emission orders reach ∼1/3 and ∼1/60 of the first-order intensity, respectively. The experimentally observed intensity ratios between the SPR bands, however, are not only dependent on the precise geometry of the metagrating, but also on the frequency dependence of the exponential decay term in eq 4. This contributes to an additional drop in excitation efficiency with increasing SPR order, as explained above. Remarkably, we note that the signals of first-order SPR on the silica and the free-space sides of the metagrating are similar in amplitude, despite attenuation of the latter in the metal coating of the fiber. Possibly, this could be attributed to multiple scattering inside the fiber, resulting in the redirection of light that is otherwise emitted under azimuthal angles that are too large to pass through the slit aperture in the HSAR imaging setup. Furthermore, numerical simulations in ref (77) have shown a 2-fold enhancement of the emission of SPR into the substrate of a silica grating that is excited by an electron beam in free space. Similarly, an enhanced local density of optical states could amplify the emission of SPR into the fiber substrate in our experiments.

**Figure 4:**
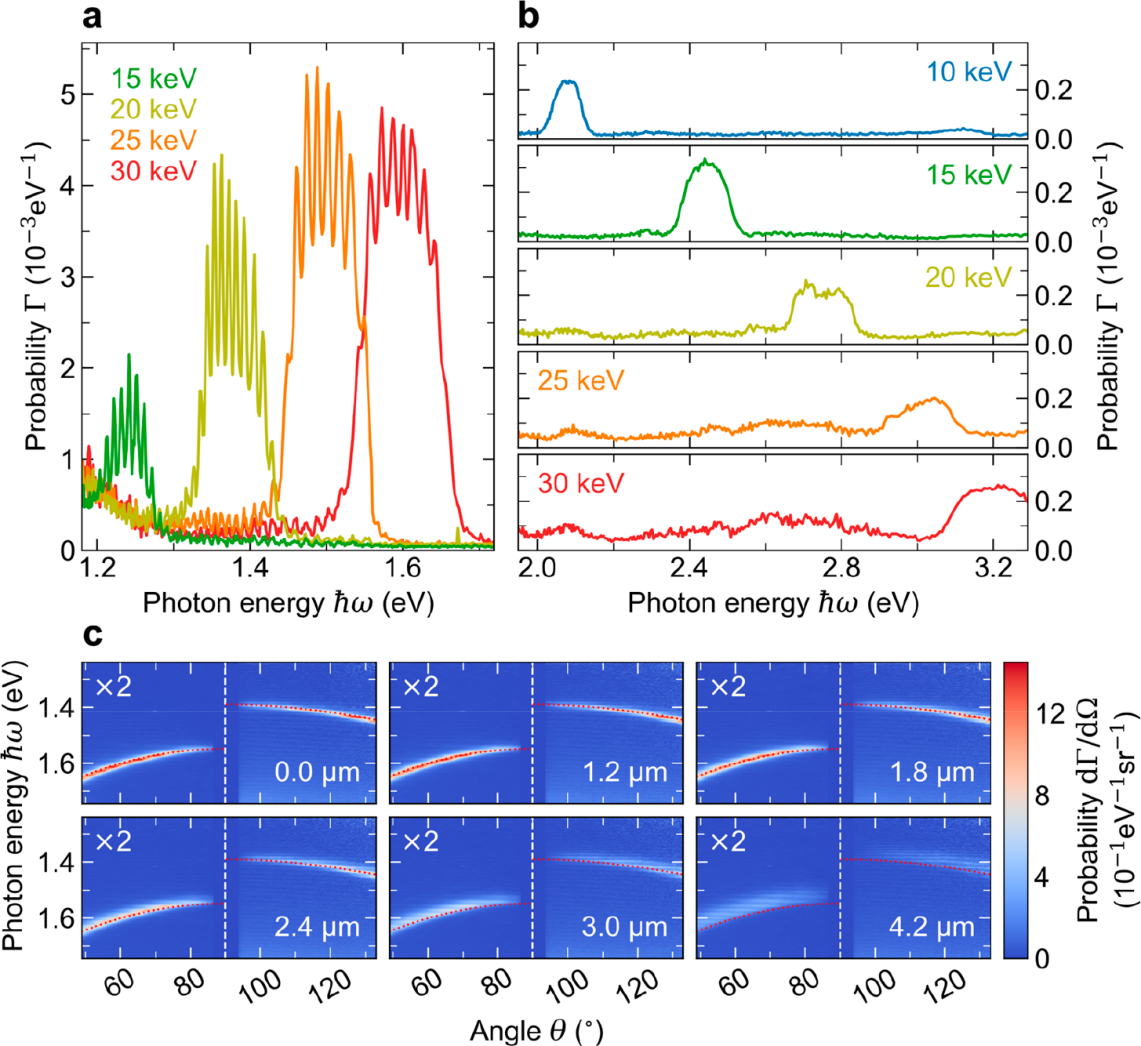
Quantitative spectral distributions of (a) first- and (b) second-order SPR that is generated on the free-space side of the metagrating as a function of the electron energy. The data are retrieved by integrating the detected HSAR far-field distributions in Figure 3 over the full range of light collection angles θ < 90°. The spectral signal oscillations in (a) occur due to an etaloning effect in the silicon sensor of the HSAR imaging setup. (c) Measured dependence of the angular dispersion of first-order SPR as collected from both sides of the metagrating on the horizontal alignment of the electron beam relative to the symmetry axis of the circular metagrating. With increasing radial distance from the center (see respective labels), the excitation efficiency of SPR decreases, the dispersion shifts to the red, and broadens spectrally.

Figure 4a,b shows the spectra of first- and second-order SPR integrated over the angular data range on the free-space side of the metagrating (θ < 90°). The measurement at 5 keV is not shown due to its low signal-to-noise ratio. The first-order SPR signals are modulated by artificial spectral oscillations due to an etaloning effect in the silicon detector at NIR frequencies. Interestingly, a downward trend in the excitation efficiency of first-order SPR is observed with decreasing electron energy (Figure 4a), whereas the excitation efficiency of second-order SPR remains rather constant (Figure 4b). A detailed theoretical discussion of how these efficiencies scale with the electron velocity is provided in ref (43). It is shown that the optimum coupling velocity strongly depends on the product of the grazing distance *d* and the exponential decay constant κ of the Fourier wave that couples to the electron. Using the approximation ,^52^ we find that for *m* = 1, ℏω = 1.6 eV, and θ = 0°, a grazing distance of *d* = 25 nm translates to κ*d* ≈ 0.76. For this situation (electron velocity β ≈ 0.26), the theory in ref (43) predicts efficient coupling of the electron to SPR, while at larger distances the coupling efficiency rapidly decreases. In our experiments, however, we note that the effective excitation strength is determined by a distribution of grazing distances due to the finite width of the electron beam, the beam convergence angle, as well as beam-sample-alignment imperfections. Since the performance of the electron optics degrades below 20 keV, this could also explain the comparatively low SPR-signal amplitude at 5 keV.

To further quantify the interaction strength between the electrons and the metagrating, we integrate over the first- and second-order SPR signals at 30 keV (spectral bandwidth ∼ 0.2 eV), yielding absolute photon emission probabilities per electron of ∼4.7 × 10^–4^ and ∼5.0 × 10^–5^, respectively. In addition, in Figure 4c, we demonstrate the effect of the horizontal electron-beam alignment relative to the symmetry axis of the circular metagrating at an electron energy of 30 keV. As the radial offset from the center is increased, we observe a consistent red-shift and spectral broadening of the first-order SPR bands in free space and in the fiber. Moreover, the signals gradually fade out in intensity. These observations demonstrate that the electron beam couples to a decreasing number of unit cells with an effective distribution of grating pitches that increase in magnitude away from the center, due to the circular symmetry of the metagrating.

**Figure 5:**
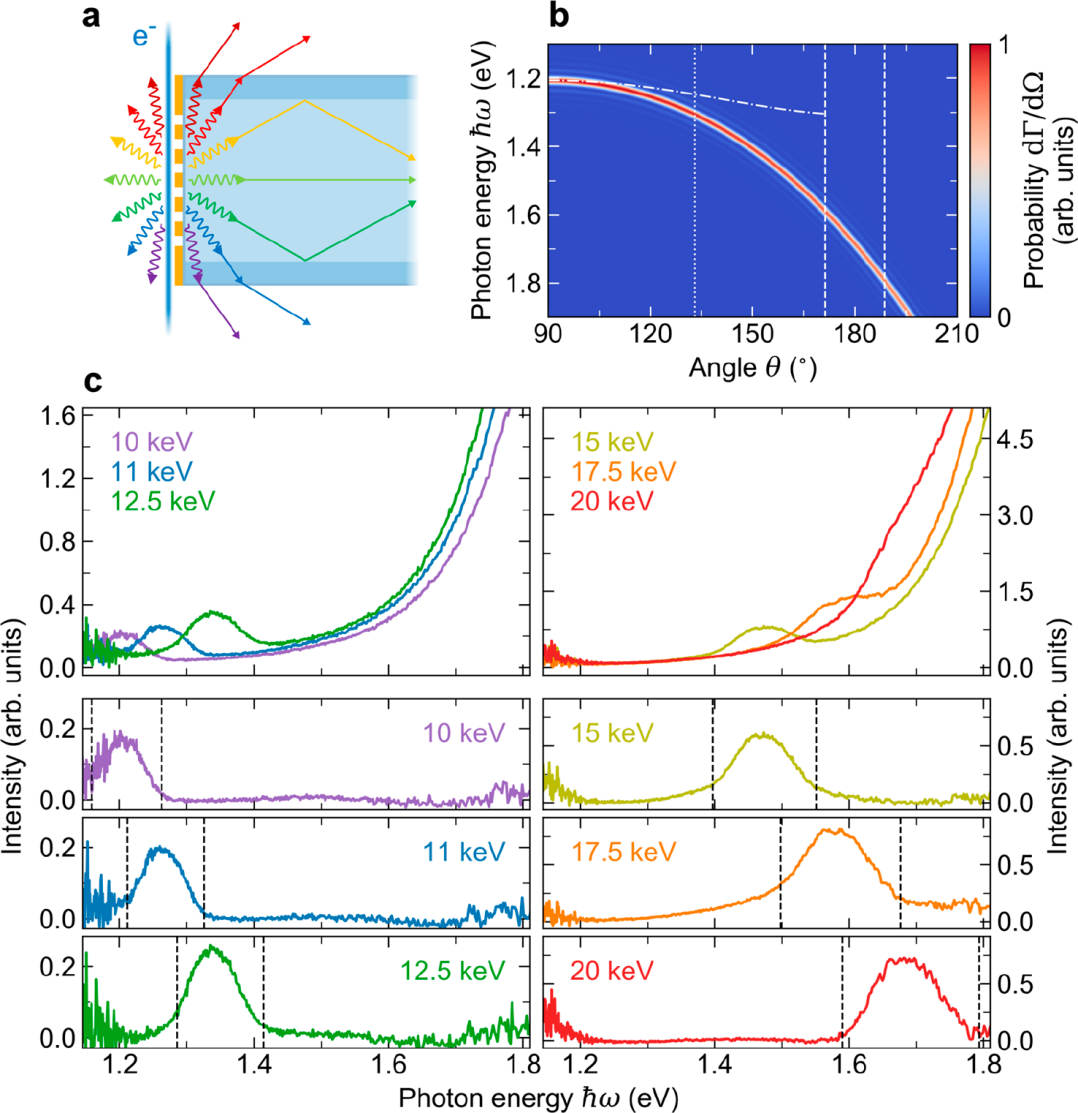
(a) Illustration of SPR that is generated in- and outside the fiber by an electron beam that excites the metagrating in free space (electron propagating downward). Light inside the fiber that is emitted under sufficiently large angles penetrates through the cladding layer and refracts at the fiber/vacuum interface. The remaining waves undergo total internal reflection at the core/cladding interface, thereby coupling to guided optical modes. (b) Analytical calculation of the spectral and angular distribution of first-order SPR that is generated on the fiber-core side of the metagrating at an electron energy of 20 keV (eq 4 with *N*_1_ = 60, *p* = 200 nm, *n* = 1.46, and *d* = 10 nm). The dashed-dotted curve shows the corresponding dispersion of SPR that leaks out of the fiber, with the dotted vertical line indicating the maximum light collection angle of the parabolic mirror in the HSAR imaging setup. The two dashed vertical lines enclose the fraction of SPR that is emitted within the NA of the fiber. (c) Large top panels: Spectral distribution of radiation collected through the fiber for excitation of the metagrating at variable electron energies between 10 and 20 keV. Small bottom panels: Extracted SPR signal as retrieved by fitting an analytical model curve to the data and subtracting the contribution of an incoherent signal background. For each electron energy, the dashed vertical lines indicate the lower and upper cutoff photon energies of SPR that is emitted within the NA of fiber as predicted by eq 1 for *m* = 1, *p* = 200 nm, and *n* = 1.46.

Finally, we turn our attention to the coupling of SPR into guided optical modes that are supported by the fiber. As illustrated in Figure 5a, photons that are emitted under sufficiently small angles relative to the optical axis of the fiber undergo total internal reflection at the core/cladding interface. This condition is determined by the NA of the fiber, which defines the maximum angle at which light can be injected through a cleaved input facet from free space. Hence, SPR that is generated on the backside of the metagrating couples to guided optical modes within an angular range of Δθ = 2arcsin(NA/*n*). For reference, Figure 5b shows an analytical calculation of the spectral and angular distribution of SPR inside the fiber induced by a 20 keV electron beam (eq 4). The vertical dashed lines enclose the component of SPR that couples into guided optical modes assuming an NA of 0.22. In addition, the dotted vertical line indicates the maximum light collection angle of the parabolic mirror in the HSAR imaging setup, and the dashed-dotted curve represents the dispersion of SPR that escapes from the fiber as described by eq 2. The left and right top panels in Figure 5c show spectra that are detected through the fiber at variable electron energies between 10 and 20 keV. The large broadband peak at the high energy end of the spectra corresponds to the tail of the NBOHC band, as discussed above. Superimposed with this band, a distinct signal contribution is observed that gradually shifts to the blue with increasing electron energy. This signal corresponds to the guided component of first-order SPR that is generated on the back side of the metagrating. To filter out the incoherent background, we again resort to a least-squares fit of an analytical model based on eq S2 in the Supporting Information and a pseudo-Voigt profile that describes the SPR signal. By subtracting the fitted incoherent signal contributions from the data, we find the spectra shown in the bottom panels in Figure 5c for the different electron energies. The dashed vertical lines indicate the cutoff photon energies that correspond to the guided fraction of SPR within the NA of the fiber according to eq 1. As can be seen, the extracted SPR signal is in excellent agreement with the calculated spectral cutoff energies, corroborating that the dispersion of SPR inside the fiber behaves as predicted by the theory.

## Discussion and Conclusion

In this work, we have studied the coupling of free electrons and light in a circular metallo-dielectric metagrating that is fabricated onto the input facet of an optical fiber. Using HSAR far-field imaging inside an SEM, we have experimentally resolved the angular dispersion of SPR that is excited on both the free-space and the fiber-core sides of the metagrating for variable electron energies between 5 and 30 keV. We observe first-, second-, and third-order emission of SPR in the UV to NIR spectral range with an angular dispersion that consistently follows the theory. Furthermore, we find good correspondence between the numerical aperture of the fiber and the spectral distribution of first-order SPR that couples into guided optical modes. Aside from these coherent excitations, we also detect a broadband distribution of incoherent defect cathodoluminescence that is generated by electrons penetrating the input facet of the fiber and scattering inelastically. Light that leaks out of the fiber is found to couple to a Rayleigh anomaly of the metagrating, giving rise to a Fano resonance. At an electron energy of 20 keV, the dispersion of this phenomenon overlaps with the emission of second-order SPR, demonstrating that the metagrating geometry could be further optimized to enhance and control the emission of SPR by exploiting resonant electromagnetic phenomena such as lattice resonances.^55,68,69^

Comparing the measured signal amplitudes, we find that the excitation strength of first-order SPR in free space is approximately 1 and 2 orders of magnitude larger than those of the second and the third SPR orders, respectively. These ratios can be ascribed to the different Fourier wave amplitudes of the SPR orders in the near-field of the metagrating as well as an exponential decay of the SPR excitation efficiency with the energy of the emitted photons. The corresponding absolute photon emission probabilities of first- and second-order SPR are found to be ∼4.7 × 10^–4^ and ∼5.0. 10^–5^, respectively (over a spectral bandwidth of ∼0.2 eV). For reference, we note that the authors of ref (43) report a theoretical maximum emission probability of SPR in the VIS spectral range of the order of 10^–4^–10^–3^ for a grating with a total length of 10 μm, a spectral bandwidth of 0.1 eV, a grazing distance of 30 nm, and 30 keV electron energy. A similar value is also obtained from simulations in the Supporting Information to ref (52). However, the calibration of the measured photon emission probabilities is optimized for CL emission processes that resemble the angular distribution of transition radiation (TR).^11,52^ In contrast, the emission of SPR is highly directional which renders an uncertainty on the absolute values of the experimental data. Furthermore, the excitation strength of SPR is highly dependent on the grazing distance of the electron beam relative to the metagrating. As mentioned above, this distance can vary substantially along the beam path due to the beam convergence angle, as well as imperfections in the beam-sample alignment. Incidentally, in ref (9) the same TR-based calibration procedure yields CL emission probabilities in the order of 10^–5^ for the excitation of plasmonic resonances in the tips of a monocrystalline gold nanostar. Here, the electron beam is targeted directly at the nanostar tips, enabling a maximum coupling efficiency between the electrons and the induced optical field. In comparison, the excitation of the metagrating is less controlled, the polycrystalline metal film might be more lossy than the nanotips, and the emission of SPR is a nonresonant phenomenon. Nevertheless, the metagrating provides a large number of unit cells that contribute to the radiation, likely explaining an overall larger probability for the emission of first-order SPR as compared to the resonant emission of CL by the nanotips.

The results presented in this work provide valuable insights into the design of future metasurface geometries that mediate the coupling between free electrons and light through an optical fiber. For example, lattice resonances based on plasmonic or dielectric structures enable efficient coupling of SPR to highly localized near-field distributions while simultaneously controlling its spectrum and polarization.^43,55,68,69^ By further introducing femto- or even attosecond electron pulses, this provides a promising pathway toward ultrafast optical-fiber-based light sources. Alternatively, high-quality-factor cavities that are coupled to an optical fiber can be used to enhance the coupling efficiency between free electrons and light within a narrow spectral range.^78^ However, due to the comparatively low spatial field confinement, such geometries are typically not suited for slow electrons in an SEM. Instead, the nanometric structure of metasurfaces provides maximal freedom to harness and engineer the SP effect. The precise arrangement of meta-atoms allows controlling the amplitude of suitable SPR orders, and can be also adapted by exploiting the refractive index contrast between the fiber substrate and free space. Vice versa, the concept of fiber-integrated metasurfaces offers great potential to mediate the coupling of light to slow free electrons, rendering an efficient platform for preparing complex free-electron quantum states. Lastly, measurements of the pulse characteristics and statistics of light collected through the fiber could reveal new insights into the correlations between optical excitations in the metasurface and their interaction with free electrons.^70^

## Data Availability

The data that support the findings of this study are available from the corresponding author upon reasonable request.

## Supporting Information Available

Analytical modeling of the incoherent signal contributions and the SPR spectral power distribution (PDF)
