# Supplementary material for: Angular Dispersion of Free-Electron-Light Coupling in an Optical Fiber-Integrated Metagrating
Source: ACS Photonics. 2024 Mar 2;11(3):1125–36. doi: 10.1021/acsphotonics.3c01574 (PMC10958598; doi:10.1021/acsphotonics.3c01574)
Supplement: Supplementary file 1 — ph3c01574_si_001.pdf [file ph3c01574_si_001.pdf]

Supporting Information: Angular dispersion of free-electron-light coupling in an optical fibre-integrated metagrating

Matthias Liebtrau and Albert Polman*

Center for Nanophotonics, NWO-Institute AMOLF
Science Park 104, 1098 XG Amsterdam, The Netherlands

*Corresponding author: a.polman@amolf.nl

S1 Analytical model of the incoherent signal contributions

To capture the Fano resonance on the free-space side of the metagrating (θ < 90°), we use an expression of the form[1]

$$F(\omega) = \frac{(q+\varepsilon)^2+b}{(1+\varepsilon^2)} G(\omega), \tag{S1}$$

with $\varepsilon = \hbar(\omega - \omega_\mathrm{f})/\gamma$ the reduced photon energy, γ the Fano linewidth, q the Fano parameter, and $G(\omega)$ the spectrum of the incoherent defect cathodoluminescence. The parameter b accounts for a flattening of the typical dip on the low energy side of the Fano resonance due to optical material losses[1]. The NBOHC and the ODC bands are modelled as a superposition of two exponentially-modified Gaussian distributions given by

$$G_i(\omega) = (A_i/2)\exp(\alpha_i \hbar(\omega - \omega_i))\,\mathrm{erfc}\left(\frac{1}{\sqrt{2}\sigma_i}\left(\alpha_i \sigma_i^2 + \hbar(\omega - \omega_i)\right)\right), \tag{S2}$$

where $\mathrm{erfc}(x)$ denotes the complementary error function, A_i is the amplitude, $\Gamma_i = 2\sqrt{2\ln(2)}\sigma_i$ is the linewidth, α_i is the asymmetry parameter, and $\hbar\omega_i$ is the mean photon emission energy of defect i. Lastly, to cover the artificial background signal due to the ODC band in the NIR spectral range (see methods section in the main text), we introduce a combination of two sigmoidal functions with a joint transition point at 1.24 eV. While this choice has no explicit physical motivation, it provides an efficient representation of the data, also in view of an elevated noise floor near the silicon band edge at 1.12 eV.

To fix the parameters $\hbar\omega_i$ and Γ_i in Eq. (S2), we perform a separate HSAR measurement at each electron energy in which the electron beam is aimed at the alignment marker in the centre of the metagrating (thereby minimizing the generation of SPR). By applying a least-squares minimization algorithm, we fit a background model to the measured spectra for all angular samples on the backside of the metagrating ($\theta > 90°$) and in the spectral data range between 1.51 eV and 3.29 eV. Thus, we obtain mean photon emission energies of $\hbar\omega_\mathrm{NBOHC} \approx$ 1.95 eV and $\hbar\omega_\mathrm{ODC} \approx$ 2.80 eV, as well as spectral line widths of $\Gamma_\mathrm{NBOHC} \approx$ 0.13 eV and $\Gamma_\mathrm{ODC} \approx$ 0.33 eV, in good agreement with values previously reported in the literature[2,3].

S2 Analytical derivation of SPR spectral power distribution

To derive the spectral power distribution of SPR that results from the coherent interaction between a point-like free electron and a grating, we resort to an approximate analytical expression as provided by Eq. S5 in the Supplementary Information to ref.[4]. Assuming the electron to propagate strictly parallel to the surface of the grating and orthogonal to the rulings at a constant grazing distance d, this expression is proportional to an integral of the form

$$S_m(\omega) \propto e^{-2\kappa d} \left| \int_{-L}^{L} \mathrm{d}z e^{-i\left(\frac{\omega}{c}(n\sin\theta+\beta^{-1})-m\frac{2\pi}{p}\right)z} \right|^2, \quad \text{(S3)}$$

where θ is defined according to the light collection angle in the main text, and $2L$ is the interaction length for an integer number of $2N$ unit cells. The parameter $\kappa \approx \frac{\omega}{c}\sqrt{\beta^{-2} - n^2}$ describes the evanescent decay of the excitation strength normal to the grating surface, with $\beta = v/c$ the normalized electron velocity. Note, that we have generalized Eq. S5 in the Supporting Information to ref.[4] to an arbitrary diffraction order m as well as the emission of SPR in a homogenous, lossless medium with refractive index n.

Since the integrand in Eq. (S3) obeys the periodicity of the grating, we can reduce the problem to an integral over a single unit cell, while taking into account the relative phase contributions of all the remaining cells in the lattice. Thus, we find

$$S_m(\omega) \propto e^{-2\kappa d} \left| \sum_{u=-N}^{(N-1)} e^{-i(\phi-2\pi m)u} \int_{-p/2}^{p/2} \mathrm{d}z e^{-i(\phi-2\pi m)\frac{z}{p}} \right|^2, \quad \text{(S4)}$$

where $\phi = \frac{\omega}{c}(n\sin\theta + \beta^{-1})p$. Further, we can exploit the geometric sum identity $\sum_{u=0}^{2N-1} \rho^{(u-N)} = \rho^{-N}(1-\rho^{2N})/(1-\rho)$ as well as the Fourier integral of the box function, $\int_{-\frac{\tau}{2}}^{\frac{\tau}{2}} \mathrm{d}t\, e^{-i2\pi f t} = \tau\,\mathrm{sinc}(f\tau)$, where τ is the box width, and $\mathrm{sinc}(x) = \sin(\pi x)/(\pi x)$ is the normalized sinc function. For $\rho = \exp\big(-i(\phi - 2\pi m)\big)$ we obtain $|\rho^{-N}(1-\rho^{2N})/(1-\rho)| = \left|\sin(N\phi)/\sin\left(\frac{1}{2}\phi\right)\right|$, which by substituting $N_m = 2N$ eventually yields the spectral lineshape of the SPR signal as

$$S_m(\omega) \propto e^{-2\kappa d} \left| \frac{\sin(N_m\phi/2)}{\sin(\phi/2)} \right|^2 \left| \mathrm{sinc}\left(m - \frac{\phi}{2\pi}\right) \right|^2. \quad \text{(S5)}$$

References

1. Gallinet, B. & Martin, O. J. F. Ab initio theory of Fano resonances in plasmonic nanostructures and metamaterials. *Phys Rev B* **83**, 235427 (2011).
2. Skuja, L. Optically active oxygen-deficiency-related centers in amorphous silicon dioxide. *J Non Cryst Solids* **239**, 16–48 (1998).
3. Fournier, J. *et al.* Luminescence study of defects in silica glasses under near-UV excitation. *Phys Procedia* **8**, 39–43 (2010).
4. Karnieli, A. *et al.* Cylindrical Metalens for Generation and Focusing of Free-Electron Radiation. *Nano Lett* **22**, 5641–5650 (2022).
